# Association of α-fetoprotein levels with liver stiffness measurement in outpatients with chronic hepatitis B

**DOI:** 10.1042/BSR20203048

**Published:** 2021-01-06

**Authors:** Juan Wang, Pengpeng Zhang, Juan Liao, Yan Zhu, Xiaoli Liu, Hong Tang

**Affiliations:** 1 Center of Infectious Diseases, West China Hospital of Sichuan University, Chengdu, China; 2 Transplantation Center, The Third Xiangya Hospital, Central South University, Changsha, Hunan, China; 3 Employee Healthcare Department, West China Hospital of Sichuan University, Chengdu, China; 4 Department of Infectious Diseases, Hunan Provincial Corps Hospital, Chinese People’s Armed Police Forces, Hunan, China

**Keywords:** AFP, chronic hepatitis B, liver fibrosis, liver stiffness measurement

## Abstract

The association between α-fetoprotein (AFP) levels with the assessment of liver stiffness (LS) in chronic hepatitis B (CHB) patients were explored. A total of 283 outpatients with CHB were enrolled. Patient age, alanine aminotransferase (ALT), aspartate aminotransferase (AST), AFP, platelet (PLT), total bilirubin (TB), direct bilirubin (DB), alkaline phosphatase (ALP), albumin (ALB), globulin, and albumin/globulin (A/G) levels were associated with LS values in the univariate model (*P*<0.05). Significant associations between AFP and PLT levels with LS values were observed when both variables were included in the multivariate analysis models. Receiver operation characteristic (ROC) analysis indicated that the combination of AFP and PLT levels could enhance the predictive performance of liver fibrosis (area under the curve (AUC) = 0.819, *P*<0.001) and that PLT levels (PLT < 100 × 10^9^/l) combined with high AFP levels (AFP > 8 ng/ml) significantly increased the prediction of liver fibrosis (OR = 11.216). More importantly, LS values associated with higher AFP levels (AFP > 8 ng/ml), independently of higher ALT or AST values, were significantly higher than those of low AFP level groups. In conclusion, in Chinese outpatients with CHB, AFP outperformed ALT and/or AST levels in terms of their association with LS. AFP and PLT levels were independently associated with LS, and their combined assessment could enhance the diagnostic and predictive performance of liver fibrosis among CHB patients.

## Introduction

Chronic hepatitis B (CHB) remains the main cause of chronic liver diseases (CLDs) worldwide, which consists of a process of progressive destruction and regeneration of liver parenchyma [1,2]. Liver fibrosis is the result of an excessive accumulation of extracellular matrix proteins caused by chronic liver damage. The management and prognosis of CLDs largely depend on the degree of liver fibrosis. Liver biopsy is an invasive and painful procedure, and has traditionally been considered the gold standard for assessing liver fibrosis. However, the risk of life-threatening complications and patient discomfort has limited the widespread application of routine liver biopsy [3,4], while its accuracy for evaluating hepatic fibrosis has also been questioned due to sampling errors and availability of clinical expertise [5,6].

Recently, the measurement of liver stiffness (LS) by transient elastography has been extensively proposed as a non-invasive tool for assessing liver fibrosis in different CLDs [7–9]. Furthermore, transient elastography has been recommended by international committees including the American Association for the Study of Liver Diseases (AASLD) and the European Association for the Study of the Liver (EASL) for the assessment of LS in CHB patients. In our previous study, we demonstrated that transient elastography and liver biopsy were reliable indicators of LS in the assessment of CHB patients [10]. Accordingly, a recent study also reported that transient elastography remained the most effective method for evaluating all degrees of fibrosis [11]. Transient elastography has been widely adopted in the clinical setting given its advantages of being a non-invasive, convenient, economic and dynamic follow-up detection approach, and for its utility in diagnosing progressive liver fibrosis and early cirrhosis without the requirement for liver biopsies in CHB patients. Previous studies have indicated that some serum indexes, such as alanine aminotransferase (ALT), aspartate aminotransferase (AST), and platelet (PLT) levels, were associated with LS values [12,13] and predictive models for liver fibrosis (APRI or FIB-4 scores) have been built based on these values [14–17]. However, to date, there have been no studies correlating serum α-fetoprotein (AFP) levels with LS measurement in patients with CHB.

AFP constitutes most of the serum proteins in the fetus and is considered a marker of hepatocellular carcinoma (HCC) and other tumors [18–20]. Previous studies have also indicated that AFP serum levels are elevated in acute and chronic hepatitis C (HCV) patients and especially in the presence of hepatic steatosis and fibrosis [21,22], and thus have been included in many surrogate biochemical scores for the prediction of different stages of hepatic fibrosis [23,24]. Hence, the aim of present study was to investigate the association of AFP levels with the degree of LS stiffness. Further, the study aimed to provide a predictive tool for determining liver fibrosis degree based on AFP levels to help clinicians more effectively identify the risk of developing fibrosis in CHB patients.

## Methods

### Study population

**Figure 1:**
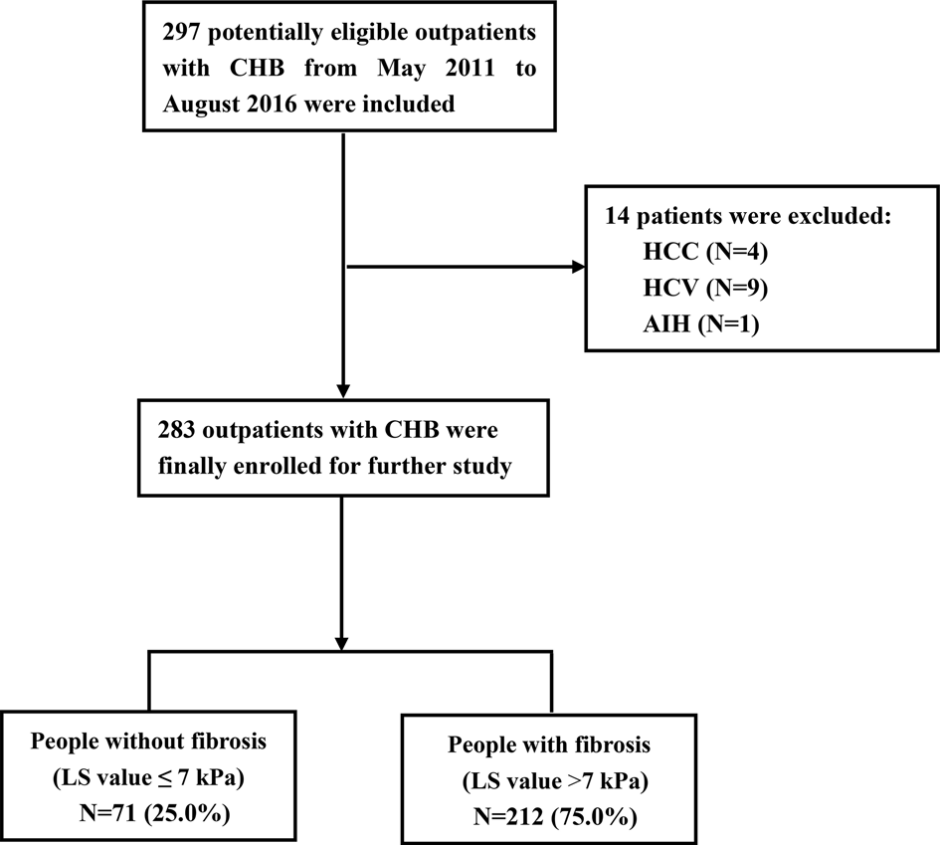
The study flowchart Abbreviation: AIH, autoimmune hepatitis.

A total of 283 outpatients with CHB were enrolled according to the flowchart shown in Figure 1. For all patients, clinical characteristics including age, sex, body mass index (BMI), ALT, AST, AFP, PLT, total bilirubin (TB), direct bilirubin (DB), mean serum albumin (ALB), globulin (GLOB), ALB/GLOB (A/G) ratio, and alkaline phosphatase (ALP) were collected at the time of LS measurement. Serum ALT, AST, TB, DB, ALB, GLOB and ALP were measured using a Cobas c702 Automatic Analyzer (Germany, U/L). Serum AFP was measured using an E170c Automatic Analyzer (Germany, U/L). PLT levels were measured using a Sysmex XS-2000i autoanalyzer (Japan). Normal values for ALT and AST ranged from 0 to 40 U/l. AFP ranged from 0 to 8 ng/ml and PLT ranged from 100 to 300 × 10^9^/l. The normal values of other variables were determined based on the manufacturer’s instructions.

### LS measurement

LS was measured by transient elastography using FibroScan (EchoSens, Paris, France); the details of the examination procedure and technical background have been described previously [25]. Briefly, the measurement depth was between 25 and 65 mm and ten successful acquisitions were performed for each patient. The success rate was calculated as the ratio of the number of successful acquisitions divided by the total number of acquisitions and only a success rate of more than 60% were considered reliable. The median value was considered representative of the liver elastic modulus and the LS value was expressed in kilopascals (kPa). In the present study, a cut-off value of 7.0 kPa was defined to identify liver fibrosis, which was based on the previous study that proposed a normal LS range of 3.3–6.8 kPa in women and 3.7–7.0 kPa in men [26]. Wong et al. proposed that 7.0 kPa could act as an optimal cut-off LS value to diagnose liver fibrosis in patients with nonalcoholic fatty liver disease [27].

### Statistical analysis

All statistical analyses were performed using SPSS version 20.0 (SPSS Inc., Chicago, IL, U.S.A.) and GraphPad Prism 8.0 (San Diego, CA, U.S.A.). Baseline characteristics are reported as mean ± standard deviation (SD) for continuous variables, or by percentage for categorical variables. Correlation analysis was used to determine the correlation between the LS values and serum AFP levels. For comparisons of continuous variables, Student’s *t* test or Mann–Whitney U-test was used, as appropriate. Chi-square analysis or Fisher’s exact tests was used for comparisons of categorical variables. Only variables with a statistically significant association (*P*<0.05) in the univariate analysis were included in the multiple logistic regression model. Multivariate logistic regression analysis was performed to test the association between different variables and LS values and all the variables were analyzed with the method ‘Backward: LR’. We assessed the discriminative power of the identified biomarkers on the prediction of liver fibrosis using receiver operation characteristic (ROC) curves and obtained the sensitivity, specificity, positive predict value (PPV), negative predict value (NPV) and area under the curve (AUC) with 95% confidence interval (CI) for each potential predictor. Out-of-Sample Validation and Hosmer–Lemeshow test were applied for validation and calibration, respectively. The comparison of new prediction models of liver fibrosis with current models was performed using MedCalc software. A *P*-value <0.05 (two-tailed) was considered statistically significant.

## Results

### General characteristics

Prescreening led to the identification of 297 eligible outpatients with CHB attending the West China Hospital, Sichuan University, Chengdu, China from May 2011 to August 2016. Fourteen patients with a diagnosis of HCC or other tumors, or who were infected with another hepatitis virus, or other causes of CLD were excluded. A total of 283 outpatients with CHB were finally enrolled (Figure 1) and all patients had undergone LS measurement. The mean ± SD of LS values and blood biomarkers are presented in Table 1. Baseline characteristics of the study participants indicated that the mean age of the enrolled patients was 39.9 ± 10.9 years. Approximately 85.2% (241) were male and the mean body BMI was 23.5 ± 2.7 kg/m^2^. The laboratory data indicated that the mean ALT and AST values were 177.9 ± 215.4 U/l and 121.5 ± 161.5 U/l, respectively. The mean AFP value was 28.1 ± 111.5 ng/ml and the mean PLT was 139.9 ± 55.9 × 10^9^/l. The mean TB and DB levels, were 21.4 ± 22.2 and 9.6 ± 19.0 µmol/l, respectively. The ALB and GLOB levels were 45.4 ± 4.7 and 30.5 ± 4.8 g/l, respectively, and the A/G ratio was 1.5 ± 0.3. The mean ALP level was 92.4 ± 31.9 IU/l. Of the 283 patients enrolled, approximately 212 (75.0%) participants presented with liver fibrosis (LS value >7.0 kPa).

**Table 1:** Demographic, laboratory and clinical variables of 283 outpatients diagnosed with CHB

Variables	Value
Demographic variables	
Age, mean years ± SD	39.9 ± 10.9
Sex, number of male (%)	241 (85.2)
BMI, mean value ± SD, kg/m^2^	23.5 ± 2.7
Laboratory variables	
ALT, U/l	177.9 ± 215.4
AST, U/l	121.5 ± 161.5
AFP, ng/ml	28.1 ± 111.5
PLT, ×10^9^/l	139.9 ± 55.9
TB, µmol/l	21.4 ± 22.2
DB, µmol/l	9.6 ± 19.0
ALB, g/l	45.4 ± 4.7
GLOB, g/l	30.5 ± 4.8
A/G	1.5 ± 0.3
ALP, IU/l	92.4 ± 31.9
Imaging variables	
LS value, kPa	14.3 ± 12.3

### Correlation of clinical factors with LS in patients with CHB

As shown in Table 2, the clinical variables were included in a univariate analysis to identify the correlation between LS values in the 283 participants with CHB. The results indicated that age, ALT, AST, AFP, PLT, TB, DB, ALB, GLOB, A/G, and ALP showed significant association with LS (*P*<0.05, Table 2). Next, 11 items were included in a multivariate model, as shown in Table 3. Significant associations for PLT and AFP levels with LS values were observed (odds ratio [OR]: 0.989, 95% CI: 0.983–0.994, *P*<0.001 and 1.354, 95% CI: 1.166–1.571, *P*<0.001, respectively).

**Table 2:** Univariate analysis: comparison of patients with and without liver fibrosis

Variables	People without fibrosis (LS value ≤ 7 kPa, 71/283, 25.0%)	People with fibrosis (LS value > 7 kPa, 212/283, 75.0%)	*P*
Age, mean years ± SD	36.7 ± 12.8	41.0 ± 10.0	0.011
Sex, number of male (%)	54/71 (76.1%)	187/212 (88.2%)	0.098
BMI, mean value ± SD, kg/m^2^	23.2 ± 2.8	23.6 ± 2.7	0.243
ALT, U/l	127.0 ± 80.6	195.01 ± 242.2	<0.001
AST, U/l	73.8 ± 41.9	137.4 ± 182.3	<0.001
AFP, ng/ml	3.3 ± 1.9	36.4 ± 127.8	<0.001
PLT, × 10^9^/l	164.6 ± 52.2	131.6 ± 54.8	<0.001
TB, µmol/l	16.5 ± 6.9	23.0 ± 25.2	0.001
DB, µmol/l	5.5 ± 2.6	10.9 ± 21.7	0.001
ALB, g/l	46.6 ± 3.9	45.0 ± 4.8	0.007
GLOB, g/l	29.0 ± 4.0	31.1 ± 5.0	0.002
A/G	1.6 ± 0.3	1.5 ± 0.3	<0.001
ALP, IU/l	82.8 ± 26.2	95.7 ± 33.0	0.001

**Table 3:** Multivariable analysis: independent factors associated with liver fibrosis (LS value > 7 kPa)

Variable	β	S.E.	OR	95% CI	*P*
AFP, ng/ml	0.303	0.076	1.354	1.166–1.571	<0.001
PLT, ×10^9^/l	-0.012	0.003	0.989	0.983–0.994	<0.001

Abbreviation: SE, standard error.

**Figure 2:**
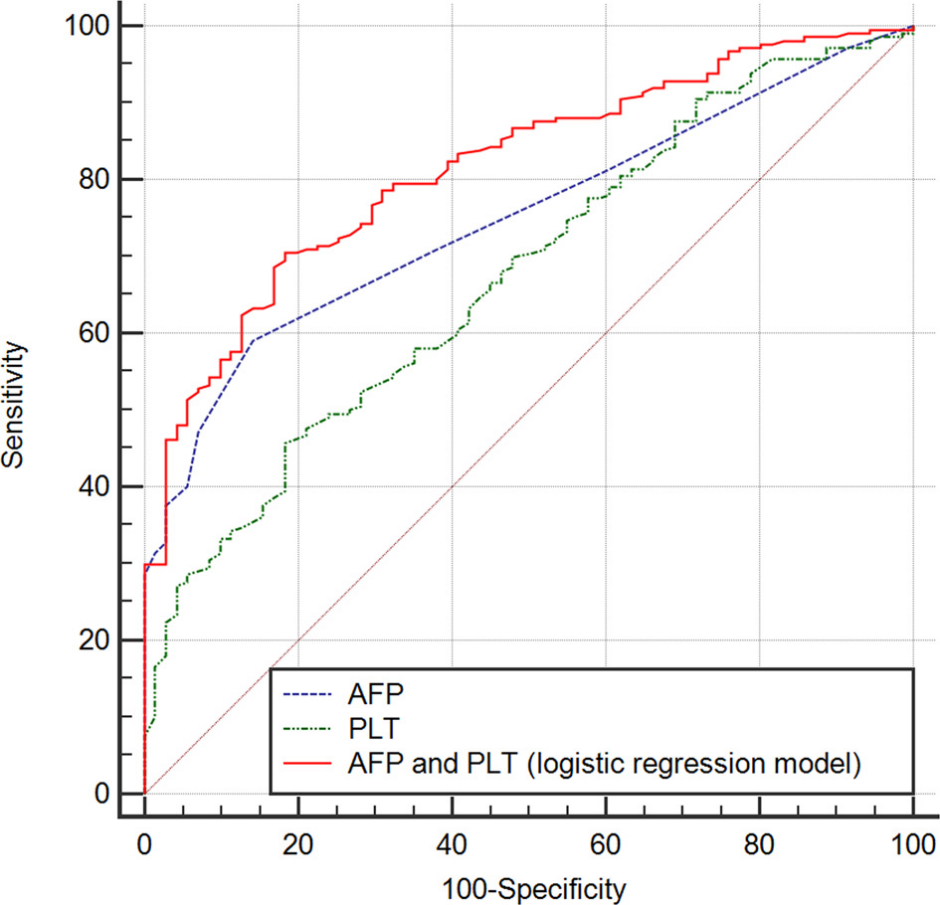
ROC curves for PLT and serum AFP levels, alone and in combination, to predict liver fibrosis (LS type> 7 kPa) The sensitivity, specificity, PPV, NPV, and AUC (95% CI and *P*-value) for PLT levels (0.460, 0.819, 86.8%, 33.7%, and 0.678 [0.611–0.746, *P*<0.001], respectively); for AFP levels (0.581, 0.873, 93.1%, 41.4%, and 0.755 [0.698–0.812, *P*<0.001], respectively); and for AFP and PLT in combination (0.682, 0.833, 92.3%, 47.2%, and 0.819 [95% CI: 0.768–0.870, *P*<0.001], respectively) were determined.

**Figure 3:**
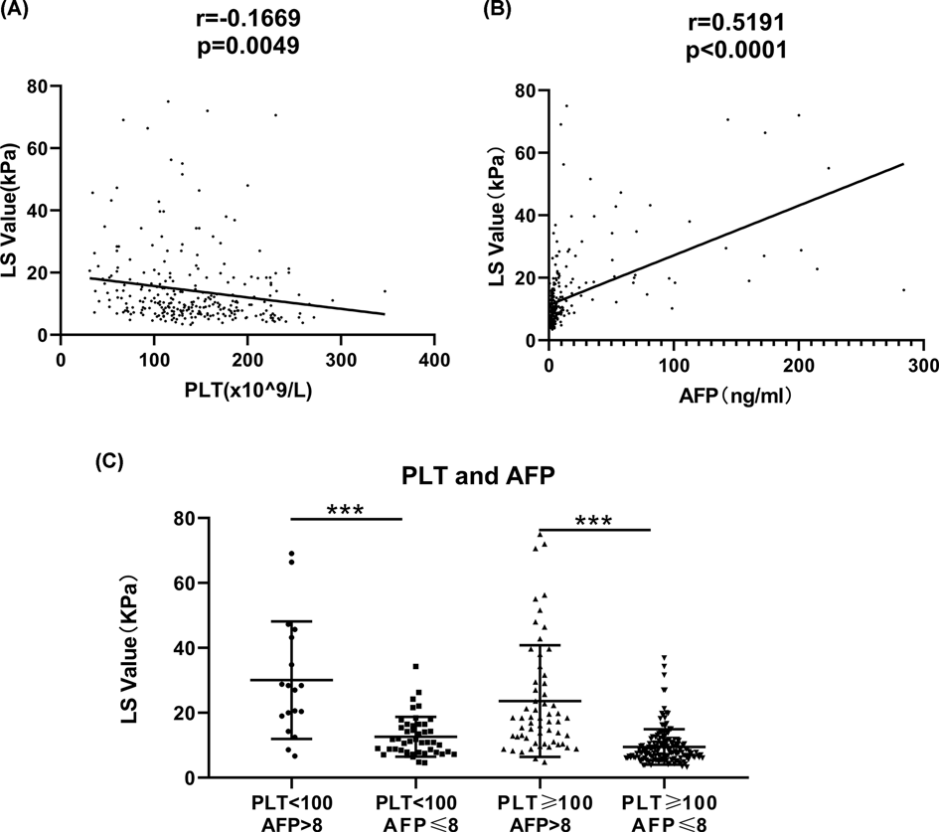
Association of PLT and AFP levels with LS values in the patients with CHB (**A**) The PLT had highly significant negative correlation with LS value. (**B**) The AFP level had highly significant positive correlation with LS value. (**C**) The LS value of high AFP level subgroups (AFP > 8 ng/ml) were increased significantly than those with low AFP level subgroups, which was not subjected to the PLT (100 × 10^9^/l).(***P<0.001).

In order to determine the effects of AFP on the diagnostic performance of LS values in patients with CHB, ROC analysis was performed. The AUC for AFP levels was 0.755 (95% CI: 0.698–0.812, sensitivity = 0.581, specificity = 0.873, PPV = 93.1%, NPV = 41.4%, *P*<0.001, Figure 2). Because of the significant association of PLT with LS values, we also evaluated the predictive performance of PLT and of the combination of these two predictors (logistic regression model: 1.285 + 0.326 × AFP [ng/ml] − 0.012 × PLT [10^9^/l]), with AUCs of 0.678 (95% CI: 0.611–0.746, sensitivity = 0.460, specificity = 0.819, PPV = 86.8%, NPV = 33.7%, *P*<0.001, Figure 2) and 0.819 (95% CI: 0.768–0.870, sensitivity = 0.682, specificity = 0.833, PPV = 92.3%, NPV = 47.2%, *P*<0.001, Figure 2), respectively. Thus, the combination of PLT and AFP enhanced the predictive performance of liver fibrosis in patients with CHB. Meanwhile, the correlation analyses indicated that PLT and AFP levels had highly significant negative (r = −0.1669, *P*=0.005) and positive (r = 0.5191, *P*<0.001) correlations with the LS value, respectively (Figure 3A,B).

To further investigate the combined effects of PLT and AFP level on LS values, PLT and AFP levels were stratified into two categories according to clinical cut-off values (100 × 10^9^/l for PLT and 8 ng/ml for AFP) (Table 4). Consistent with the regression analysis results, the presence of thrombocytopenia (PLT < 100 × 10^9^/l) or high AFP levels (AFP > 8 ng/ml) improved the prediction of liver fibrosis, (OR = 6.763, 95% CI: 2.310–19.797, *P*<0.001 and OR = 18.804, 95% CI: 4.433–79.757, *P*<0.001), respectively (Table 4). As expected, thrombocytopenia (PLT < 100 × 10^9^/l) combined with a high AFP level (AFP > 8 ng/ml) also significantly increased prediction of liver fibrosis (OR = 11.216, 95% CI: 1.457–86.377, *P*=0.020, Table 4). At the same time, we analyzed the LS values among the four subgroups and the results showed that the LS values in subgroups with high AFP level (AFP > 8 ng/ml) were significantly higher than those with low AFP level subgroups (Figure 3C).

**Table 4:** The combined effects of PLT, ALT, AST, and AFP on the incidence of liver fibrosis (LS value > 7 kPa)

Variable	Case	Univariate	
		OR (95% CI)	*P*
Combination of AFP and PLT (×10^9^/l)			
Low AFP (≤8 ng/ml) and PLT ≥ 100	161	1.000 (reference)	-
Low AFP (≤8 ng/ml) and PLT < 100	45	6.763 (2.310–19.797)	<0.001
High AFP (>8 ng/ml) and PLT ≥ 100	59	18.804 (4.433–79.757)	<0.001
High AFP (>8 ng/ml) and PLT < 100	18	11.216 (1.457–86.377)	0.020
Combination of AFP and ALT			
Low AFP (≤8 ng/ml) and low ALT (≤2 ULN)	90	1.000 (reference)	-
Low AFP (≤8 ng/ml) and high ALT (>2 ULN)	117	0.749 (0.419–1.341)	0.331
High AFP (>8 ng/ml) and low ALT (≤2 ULN)	21	9.032 (1.154–70.679)	0.036
High AFP (>8 ng/ml) and high ALT (>2 ULN)	55	11.968 (2.723–52.609)	0.001
Combination of AFP and AST			
Low AFP (≤8 ng/ml) and low AST (≤2 ULN)	142	1.000 (reference)	-
Low AFP (≤8 ng/ml) and high AST (>2 ULN)	64	1.038 (0.558–1.929)	0.907
High AFP (>8 ng/ml) and low AST (≤2 ULN)	24	5.978 (1.350–26.471)	0.019
High AFP (>8 ng/ml) and high AST (>2 ULN)	53	28.261 (3.792–210.596)	0.001

Abbreviation: ULN, upper limit of normal.

**Figure 4:**
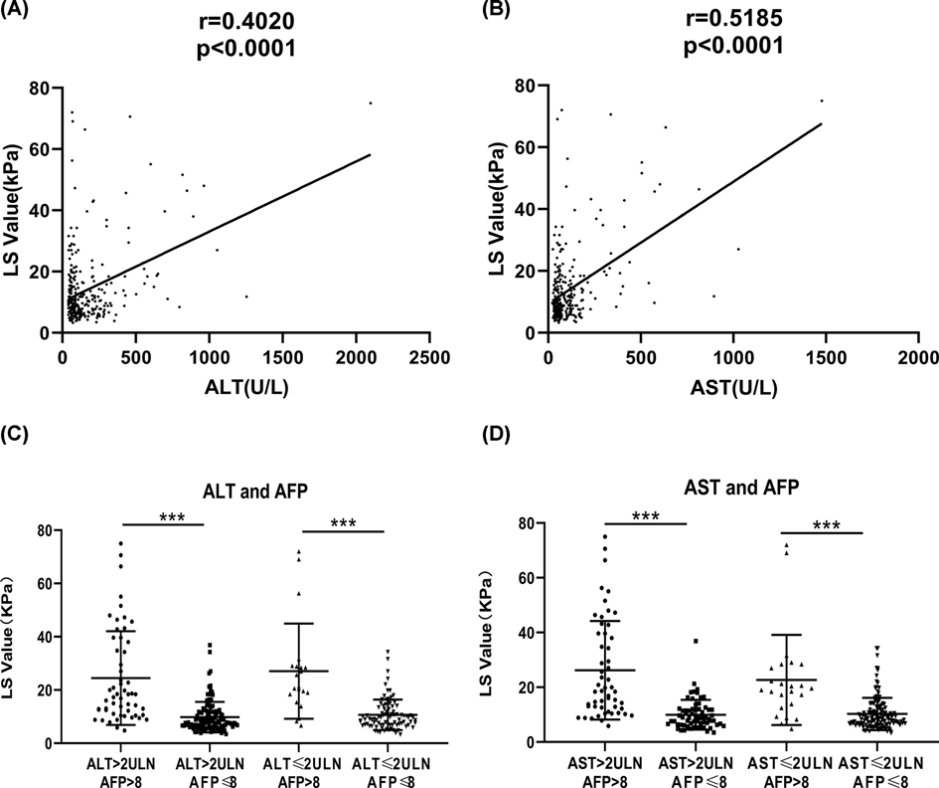
Association of ALT, AST, and AFP levels with the LS value in patients with CHB A total of 283 participants were classified according to clinical cut-off values (2 ULN for ALT and AST, 8 ng/ml for AFP). (**A,B**) The ALT and AST levels had a highly significant positive correlation with LS value. (**C,D**) The LS values of the high AFP level subgroups (AFP > 8 ng/ml), independent of whether ALT or ALT values were elevated or not, increased significantly compared with those with low AFP among outpatients with CHB (***P<0.001).

Previous studies indicated that ALT and AST levels were independently associated with the LS values [28,29]. Thus, in our univariate analyses, we compared the impact of AFP, AST, and ALT on LS values in detail. First, the correlation analyses showed that ALT and AST both had highly significant positive correlation with LS values (Figure 4A,B). Next, ALT and AST were classified as two categories according to the 2 upper limit of normal (ULN) cut-off value as in a previous study [28]. The results showed that high AFP levels (AFP > 8 ng/ml) combined with a high ALT level (ALT > 2 ULN) could significantly enhance the prediction of liver fibrosis (OR = 11.968; 95% CI: 2.723–52.609; *P*=0.001, Table 4) and high AFP level (AFP > 8 ng/ml) combined with high AST level (AST > 2 ULN) also significantly enhanced the predictive performance of liver fibrosis (OR = 28.261; 95% CI: 3.792–210.596; *P*=0.001, Table 4). In addition, the LS value in high AFP level subgroups (AFP > 8 ng/ml) significantly improved the predictive ability of liver fibrosis compared with the low AFP level subgroups, which was not subjected to the ALT or AST values (Figure 4C,D).

Finally, validation and calibration studies were also performed and the results are reported in the Supplementary File (Supplementary Figures S1 and S2).

### Comparison of the combination AFP and PLT with the current prediction model of liver fibrosis (APRI and FIB-4)

**Figure 5:**
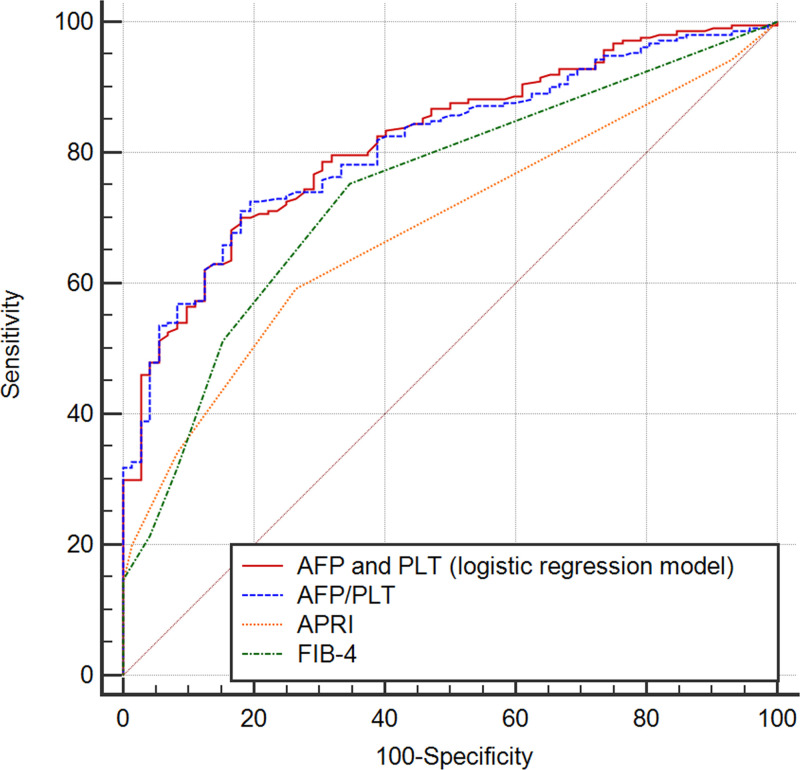
ROC curves of models tested for prediction of liver fibrosis (LS > 7 kPa) The predictive model established in our study based on logistic model (‘AFP and PLT’ logistic regression model) and the simplified score (‘AFP/PLT’) were compared with currently available prediction scores (APRI and FIB-4) by using MedCalc software.

We compared the predictive model established in our study (*1.285 + 0.326 × AFP [ng/ml] − 0.012 × PLT [10^9^/l]*) with currently available prediction scores, such as APRI and FIB-4, using MedCalc software (Figure 5 and Table 5). The results showed that our model had a higher AUC (0.819, 95% CI: 0.768–0.870) than APRI (AUC = 0.711, 95% CI: 0.648–0.775, *P*<0.001) or FIB-4 (AUC = 0.764, 95% CI: 0.701–0.825, *P*=0.018).

**Table 5:** The specific parameters of the five different prediction models

Variables	AUC (95% CI)	Sensitivity	Specificity	Cut-off value	PPV	NPV	*P* for diagnosis	*P* for comparison with AFP and PLT^*^	*P* for comparison with AFP/PLT
AFP and PLT^*^	0.819 (0.768–0.870)	0.682	0.833	1.040	92.3%	47.2%	<0.001	/	0.435
AFP/PLT	0.812 (0.761–0.863)	0.725	0.806	2.800	80.6%	50.0%	<0.001	0.435	/
APRI	0.711 (0.648–0.775)	0.673	0.694	1.280	86.6%	42.0%	<0.001	<0.001	<0.001
FIB-4	0.763 (0.701–0.825)	0.716	0.750	1.800	89.3%	47.4%	<0.001	0.018	0.042

* Logistic regression model (1.285 + 0.326 × AFP [ng/ml] − 0.012 × PLT [10^9^/l]).

In order to establish a simplifier predictive score for daily practice, ‘AFP/PLT’ score (AUC = 0.812, 95% CI: 0.761–0.863) were established. The simplified ‘AFP/PLT’ score showed no difference to previous model (difference between AUC = 0.007, 95% CI: −0.010 to 0.023, *P*=0.435, *P-values stand for* comparison between the simplified score and original score based on logistic model).

The simplified prediction models also showed better predictive value in comparison with APRI or FIB-4 scores (APRI, difference between AUC = 0.127, 95% CI: 0.066–0.189, *P*<0.001; FIB-4, difference between AUC = 0.069, 95% CI: 0.002–0.136, *P*=0.042, respectively).

## Discussion

The main etiology of liver fibrosis is chronic HBV infection in China, and thus timely and precise diagnosis of liver fibrosis is essential for the prevention and treatment of CLD [30]. LS measurement by transient elastography, as a safe and more tolerable method, has become an alternative to liver biopsy to determine the severity of liver fibrosis and cirrhosis, especially for outpatients. To the best of our knowledge, LS measurements may be influenced by different factors, however, there are no studies correlating serum AFP levels with LS measurement in patients with CHB [29,31,32].

Besides its role in HCC diagnosis, higher serum AFP levels are also observed in patients with hepatitis and liver cirrhosis [33–35]. Higher serum AFP levels have been reported to correlate significantly with advanced liver fibrosis and cirrhosis [12,36]. High levels of AFP are produced by hepatic progenitor cells in the periportal region of the liver, and AFP was determined to be responsible for liver regeneration and directly correlated with fibrosis stages [37]. Besides, a study reported that AFP could stimulate expression of epithelial cell adhesion molecule (EpCAM) [38,39], while silencing of EpCAM could suppress hepatic fibrosis and hepatic stellate cell proliferation in a mouse model of acholic hepatitis [38], indicating increased AFP level possibly led to progression of liver fibrosis by stimulating the EpCAM.

In the present study, the main finding was that both AFP and PLT levels are independently associated with LS values and their combination could improve the prognostic prediction of liver fibrosis in CHB patients (AUC = 0.819). Though AFP is mainly associated with the presence of HCC and other tumors [18–20,39], our findings indicated there was a significant positive correlation between AFP levels and LS values. Furthermore, our results are in agreement with previous studies indicating a positive correlation between AFP levels and the stage of hepatic fibrosis in chronic HCV patients without HCC [40], in addition to studies reporting that elevated serum AFP levels were independently associated with advanced stages of liver fibrosis [41]. In the present study, PLT levels were also shown to directly correlate with increased LS values. It is widely known that the hypersplenism observed in liver fibrosis is associated with thrombocytopenia [14]; thus, PLT counts have traditionally always plays an important role in the prediction of hepatic fibrosis. The FIB-4 score, includes PLT counts in its calculation, and has become a routinely available tool for the prediction of hepatic fibrosis [42]. Compared with AFP levels, PLT counts showed less predictive capability in this study (AUC = 0.755 for AFP and AUC = 0.678 for PLT), but their combination was recommended to improve discriminative power (AUC = 0.819). Furthermore, thrombocytopenia (i.e., PLT < 100 × 10^9^/l) combined with high AFP levels (AFP > 8 ng/ml) resulted in a significantly increased risk of liver fibrosis (OR = 11.216) and represents an easy-to-use method to help clinicians identify more patients at higher risk for liver fibrosis, especially among outpatients. Moreover, the model established in our study based on logistic model and the simplified score (combined AFP and PLT) also showed higher predictive value in comparison with currently available prediction scores including APRI and FIB-4. Thus, our predictive model combining thrombocytopenia and high AFP levels could have potential clinical application in the future.

In general, serum ALT and AST levels usually reflect the histologic necroinflammation status of the liver tissue and are closely associated with hepatic fibrosis. Several previous studies have indicated that higher ALT levels were associated with higher LS values [25,28,29,43]; however, Wong et al*.* reported that increased ALT was not necessarily associated with more severe hepatic fibrosis especially in acute hepatitis patients [31].

In the present study, the multivariate logistic regression analyses showed that ALT and AST level did not significantly associate with the LS value. However, when we compared the impact of AFP combined with AST, and ALT on LS, the results showed that AFP level (AFP > 8 ng/ml) and high ALT/AST levels (ALT/AST > 2 ULN) predicted a significantly increased risk of liver fibrosis (OR = 11.968 for ALT and OR = 28.261 for AST). More importantly, when participants in the study were stratified into four subgroups (Table 4), the LS value of the high AFP level subgroups (AFP > 8 ng/ml), independently of whether the ALT or AST values were increased or not, were more significantly associated with LS than patient subgroups having lower AFP levels. To the best of our knowledge, there have been no previous studies comparing the performance of these three variables on prediction of liver fibrosis. Our study indicated that serum AFP level was more reliable and effective than ALT or AST for the assessment of hepatic fibrosis progression in CHB patients. Hence, we recommend the use of AFP to predict liver fibrosis at the time of severe acute exacerbation of CHB, especially in the outpatients.

One limitation of this retrospective study is that AFP was tested at only one time point. Generally, circulating AFP levels of a patient fluctuate over time. Thus, it would be optimal to have AFP values tested at several time points in order to calculate the average AFP value for a single patient. This will be considered in future prospective studies.

## Conclusion

In conclusion, in Chinese outpatients with CHB, AFP outperforms ALT and/or AST in terms of their associations with LS, a useful tool for assessing liver fibrosis progression. PLT and AFP level were independently associated with LS, and their combination may improve the diagnostic performance and prediction of liver fibrosis. The findings could help clinicians more easily and effectively to identify CHB outpatients at the risk of developing hepatic fibrosis.

## Supplementary Material

Supplementary Figures S1-S2

## Data Availability

The datasets used and analyzed in the present study are available upon reasonable request.

## Abbreviations

AFP: α-fetoprotein
ALB: albumin
ALP: alkaline phosphatase
ALT: alanine aminotransferase
APRI: aspartate aminotransferase-to-platelet ratio index
AST: aspartate aminotransferase
AUC: area under the curve
A/G: albumin/globulin
BMI: body mass index
CHB: chronic hepatitis B
CI: confidence interval
CLD: chronic liver disease
DB: direct bilirubin
EpCAM: epithelial cell adhesion molecule
FIB-4: fibrosis 4 score
GLOB: globulin
HCC: hepatocellular carcinoma
HCV: chronic hepatitis C
LS: liver stiffness
NPV: negative predict value
OR: odds ratio
PLT: platelet
PPV: positive predict value
ROC: receiver operation characteristic
SD: standard deviation
TB: total bilirubin
ULN: upper limit of normal
