# Supplementary material for: Association of α-fetoprotein levels with liver stiffness measurement in outpatients with chronic hepatitis B
Source: Biosci Rep. 2021 Jan 6;41(1):BSR20203048. doi: 10.1042/BSR20203048 (PMC7789808; doi:10.1042/BSR20203048)
Supplement: Supplementary Figures S1-S2 [file BSR-2020-3048_supp.pdf]

1. Validation

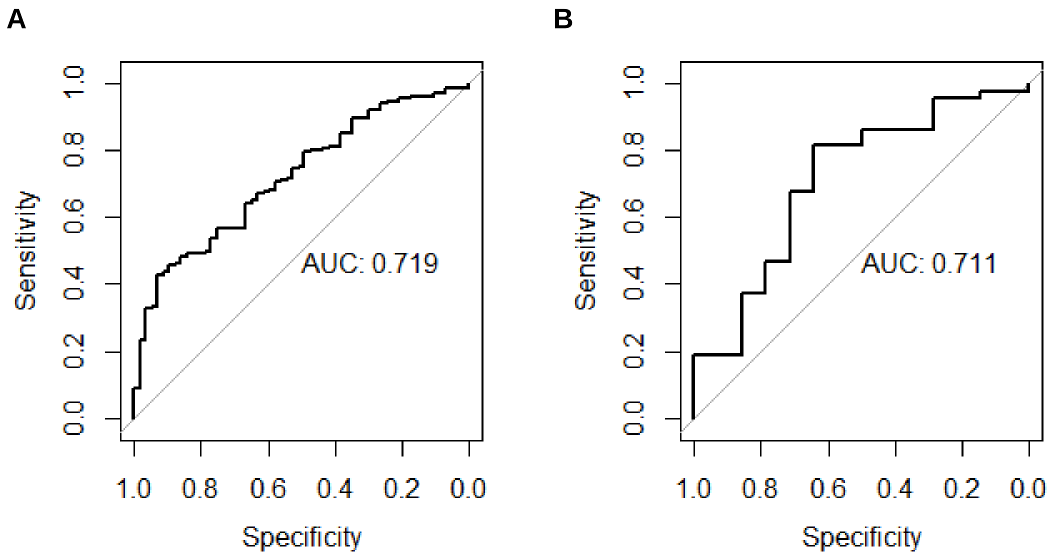

Figure S1 Out-of-Sample Validation Analysis The left one **(A)** is based on training data(80%) and the right one **(B)** is based on testing data (20%). The AUC of two figures are almost similar(0.719 V.S. 0.711).

2. Calibration

A

Hosmer-Lemeshow Test

Step	Chi-square	df	Sig.
1	8.211	8	.413
2	7.600	8	.474
3	11.669	8	.167
4	16.795	8	.032
5	11.355	8	.182
6	5.474	8	.706
7	7.383	8	.496
8	9.389	8	.311
9	6.897	8	.548

B

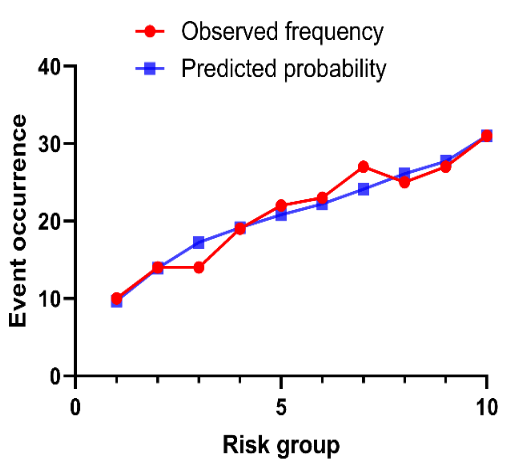

Figure S2 (A) Hosmer-Lemeshow test valuated the calibration capability of the prediction model. The result indicated that there was no significant different between the predicted value and the actual observed value （χ^2 =6.897, P>0.05） and this predicted model had a good calibration ability.**(B)**The prediction probability of each research objectin Contingency Table for Hosmer-Lemeshow Test was sorted from small to large and divided into 10 groups according to decile.And each groupof actual observations (Observed) and model prediction (Expected) were listed respectively and connected by smooth line segments in turn.The model predicted curve is close to the actual observed curve, which indicates that the calibration ability of the model is good.
